# 1-[4-(Difluoromethoxy)phenyl]-*N*-(2,3-dimethylphenyl)-1*H*-1,2,4-triazole-3-carboxamide

**DOI:** 10.1107/S160053680901263X

**Published:** 2009-04-10

**Authors:** Yu-Guang Wang, Guo-Bo Huang, Bing-Chun Zhu

**Affiliations:** a College of Biological and Environmental Engineering, Zhejiang University of Technology, Hangzhou 310014, People’s Republic of China; b School of Pharmaceutial and Chemical Engineering, Taizhou University, Taizhou 317000, People’s Republic of China; c Zhejiang University of Technology, Hangzhou 310014, People’s Republic of China; d Zhejiang Research Institute of Chemical Industry, Hangzhou 310023, People’s Republic of China

## Abstract

In the mol­ecule of the title compound, C_18_H_16_F_2_N_4_O_2_, the 1,2,4-triazole ring forms dihedral angles of 3.6 (2) and 14.9 (6)° with the 4-difluoro­meth­oxy-substituted benzene ring and the 2,3-dimethyl-substituted benzene ring, respectively. The OCHF_2_ group is twisted away from the plane of the benzene ring, as shown by the C—O—C—C torsion angle of 145.8 (2)°. The conformation is stabilized by an inter­molecular N—H⋯N hydrogen bond. In the crystal, short C—H⋯O inter­actions lead to chains of mol­ecules.

## Related literature

For general background regarding the biological and pharmacological activities of 1,2,4-triazoles and their derivatives, see: Wahbi *et al.* (1995 ▶); Chai *et al.* (2003 ▶); Hashimoto *et al.* (1990 ▶); Kalluraya *et al.* (1996 ▶); Almasirad *et al.* (2004 ▶); Amir & Shikha (2004 ▶); Kanazawa *et al.* (1988 ▶); Vlasova *et al.* (1971 ▶); Labanauskas *et al.* (2004 ▶); Tozkoparan *et al.* (2007 ▶). For a related synthesis, see: Drutkowski *et al.* (2002 ▶); Frohberg *et al.* (2002 ▶).
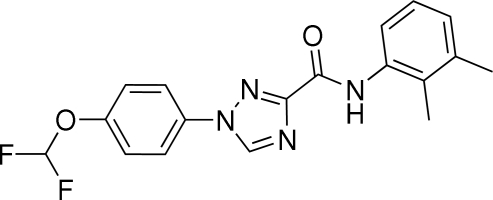


## Experimental

### 

#### Crystal data

C_18_H_16_F_2_N_4_O_2_
*M* *_r_* = 358.35
Triclinic, 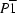

*a* = 7.5543 (10) Å
*b* = 7.8132 (10) Å
*c* = 14.8190 (19) Å
α = 95.974 (2)°
β = 98.593 (1)°
γ = 101.523 (1)°
*V* = 839.28 (19) Å^3^
*Z* = 2
Mo *K*α radiation
μ = 0.11 mm^−1^
*T* = 296 K
0.49 × 0.31 × 0.10 mm

#### Data collection

Bruker APEXII CCD diffractometer
Absorption correction: multi-scan (*SADABS*; Bruker, 2004 ▶) *T*_min_ = 0.948, *T*_max_ = 0.990
6446 measured reflections
3115 independent reflections
2366 reflections with *I* > 2σ(*I*)
*R*_int_ = 0.017

#### Refinement

*R*[*F* ^2^ > 2σ(*F* ^2^)] = 0.042
*wR*(*F* ^2^) = 0.123
*S* = 1.06
3115 reflections
238 parameters
H-atom parameters constrained
Δρ_max_ = 0.23 e Å^−3^
Δρ_min_ = −0.21 e Å^−3^

### 

Data collection: *APEX2* (Bruker, 2004 ▶); cell refinement: *SAINT* (Bruker, 2004 ▶); data reduction: *SAINT*; program(s) used to solve structure: *SHELXS97* (Sheldrick, 2008 ▶); program(s) used to refine structure: *SHELXL97* (Sheldrick, 2008 ▶); molecular graphics: *SHELXTL* (Sheldrick, 2008 ▶); software used to prepare material for publication: *SHELXTL* and *PLATON* (Spek, 2009 ▶).

## Supplementary Material

Crystal structure: contains datablocks wyg, I. DOI: 10.1107/S160053680901263X/ez2162sup1.cif

Structure factors: contains datablocks I. DOI: 10.1107/S160053680901263X/ez2162Isup2.hkl

Additional supplementary materials:  crystallographic information; 3D view; checkCIF report

## Figures and Tables

**Table 1:** Hydrogen-bond geometry (Å, °)

*D*—H⋯*A*	*D*—H	H⋯*A*	*D*⋯*A*	*D*—H⋯*A*
N4—H4*D*⋯N3	0.86	2.27	2.717 (2)	113
C6—H6⋯O2^i^	0.93	2.43	3.344 (2)	169
C8—H8⋯O2^i^	0.93	2.26	3.159 (2)	162

Symmetry code: (i) 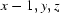.

**Fig. 1.**
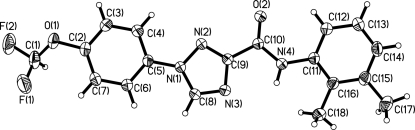
View of the title compound showing the atom numbering scheme and the ellipsoids at the 50% probability level.

## supplementary crystallographic information

### Comment

1,2,4-Triazoles and their derivatives have long been known to exhibit diverse biological and pharmacological activities, such as antitubercular, anticancer (Vlasova *et al.*, 1971; Kalluraya *et al.*, 1996), anticonvulsant (Almasirad *et al.*, 2004; Kanazawa *et al.*, 1988; Chai *et al.*, 2003; Hashimoto *et al.*, 1990), anti-inflammatory (Labanauskas *et al.* ,2004), herbicidal, and analgesic properties (Tozkoparan *et al.*, 2007; Amir & Shikha, 2004). Also, antifungal activity of aromatic ethers possessing a 1*H*-1,2,4-triazole ring has been reported (Wahbi *et al.*, 1995). Herein, we report the synthesis and crystal structure of the title compound, (I).

In the molecule of the title compound (Fig. 1) the bond lengths and angles are generally within normal ranges. The planar 1,2,4-triazole ring is oriented at dihedral angles of 3.6 (2)° and 14.9 (6)° with respect to the 4-difluoromethoxy-substituted benzene ring and 2,3-dimethyl-substituted benzene ring, respectively. The CHF_2_ group is twisted away from the plane of the benzene ring, as shown by the C1—O1—C2—C3 torsion angle [145.8 (2)°].

### Experimental

The general procedure to synthesize the title compound: 2-amine-*N*-(2,3-dimethyl-phenyl)-2-[(4-difluoromethoxy- phenyl)hydrazono]acetamide (10 mmol), 1.5 mL of a 37%-solution of formaldehyde (20 mmol) and 0.1 g *p*-toluene sulfonic acid were refluxed in approximately 50 mL ethanol. The reaction was complete after 10 h. The mixture was cooled to room temperature and the solvent was evaporated. The solid product was collected and recrystallized from 2-propanol (Drutkowski *et al.*, 2002; Frohberg *et al.*, 2002).

### Refinement

H atoms were placed in calculated positions with C—H = 0.95–0.99 Å, and refined in riding mode with *U*_iso_(H) = 1.2*U*_eq_(C).

### Crystal data

**Table:**

C_18_H_16_F_2_N_4_O_2_	*V* = 839.28 (19) Å^3^
*M**_r_* = 358.35	*Z* = 2
Triclinic, *P*1	*F*(000) = 372
*a* = 7.5543 (10) Å	*D*_x_ = 1.418 Mg m^−^^3^
*b* = 7.8132 (10) Å	Mo *K*α radiation, λ = 0.71073 Å
*c* = 14.8190 (19) Å	µ = 0.11 mm^−^^1^
α = 95.974 (2)°	*T* = 296 K
β = 98.593 (1)°	Block, white
γ = 101.523 (1)°	0.49 × 0.31 × 0.10 mm

### Data collection

**Table:**

Bruker APEXII CCD diffractometer	3115 independent reflections
Radiation source: fine-focus sealed tube	2366 reflections with *I* > 2σ(*I*)
graphite	*R*_int_ = 0.017
φ and ω scans	θ_max_ = 25.5°, θ_min_ = 2.7°
Absorption correction: multi-scan (*SADABS*; Bruker, 2004)	*h* = −9→9
*T*_min_ = 0.948, *T*_max_ = 0.990	*k* = −9→9
6446 measured reflections	*l* = −17→17

### Refinement

**Table:**

Refinement on *F*^2^	Secondary atom site location: difference Fourier map
Least-squares matrix: full	Hydrogen site location: inferred from neighbouring sites
*R*[*F*^2^ > 2σ(*F*^2^)] = 0.042	H-atom parameters constrained
*wR*(*F*^2^) = 0.123	*w* = 1/[σ^2^(*F*_o_^2^) + (0.0593*P*)^2^ + 0.1647*P*] where *P* = (*F*_o_^2^ + 2*F*_c_^2^)/3
*S* = 1.06	(Δ/σ)_max_ < 0.001
3115 reflections	Δρ_max_ = 0.23 e Å^−^^3^
238 parameters	Δρ_min_ = −0.21 e Å^−^^3^
0 restraints	Extinction correction: *SHELXL*, Fc^*^=kFc[1+0.001xFc^2^λ^3^/sin(2θ)]^-1/4^
Primary atom site location: structure-invariant direct methods	Extinction coefficient: 0.016 (3)

### Special details

**Table:**

Geometry. All e.s.d.'s (except the e.s.d. in the dihedral angle between two l.s. planes) are estimated using the full covariance matrix. The cell e.s.d.'s are taken into account individually in the estimation of e.s.d.'s in distances, angles and torsion angles; correlations between e.s.d.'s in cell parameters are only used when they are defined by crystal symmetry. An approximate (isotropic) treatment of cell e.s.d.'s is used for estimating e.s.d.'s involving l.s. planes.
Refinement. Refinement of *F*^2^ against ALL reflections. The weighted *R*-factor *wR* and goodness of fit *S* are based on *F*^2^, conventional *R*-factors *R* are based on *F*, with *F* set to zero for negative *F*^2^. The threshold expression of *F*^2^ > σ(*F*^2^) is used only for calculating *R*-factors(gt) *etc*. and is not relevant to the choice of reflections for refinement. *R*-factors based on *F*^2^ are statistically about twice as large as those based on *F*, and *R*- factors based on ALL data will be even larger.

### Fractional atomic coordinates and isotropic or equivalent isotropic displacement parameters (Å^2^)

**Table:**

	*x*	*y*	*z*	*U*_iso_*/*U*_eq_	
F1	−0.9182 (2)	0.3392 (2)	0.14577 (12)	0.1146 (6)	
F2	−0.8560 (2)	0.1332 (2)	0.05859 (11)	0.1113 (6)	
O1	−0.6301 (2)	0.3298 (2)	0.13688 (9)	0.0742 (4)	
O2	0.41124 (16)	0.80678 (19)	0.54210 (9)	0.0634 (4)	
N1	−0.15235 (18)	0.67315 (18)	0.45595 (9)	0.0458 (4)	
N2	0.03287 (19)	0.69741 (19)	0.46115 (10)	0.0472 (4)	
N3	−0.0355 (2)	0.8275 (2)	0.59091 (11)	0.0663 (5)	
N4	0.32482 (19)	0.9655 (2)	0.66010 (10)	0.0513 (4)	
H4D	0.2285	0.9901	0.6779	0.062*	
C1	−0.7981 (3)	0.2345 (3)	0.13967 (17)	0.0766 (7)	
H1	−0.7944	0.1632	0.1903	0.092*	
C2	−0.5184 (3)	0.4106 (2)	0.21999 (13)	0.0541 (5)	
C3	−0.3332 (3)	0.4212 (3)	0.22448 (13)	0.0611 (5)	
H3	−0.2899	0.3708	0.1749	0.073*	
C4	−0.2118 (3)	0.5063 (3)	0.30237 (13)	0.0556 (5)	
H4	−0.0866	0.5130	0.3057	0.067*	
C5	−0.2770 (2)	0.5815 (2)	0.37546 (11)	0.0444 (4)	
C6	−0.4633 (2)	0.5707 (2)	0.37090 (13)	0.0539 (5)	
H6	−0.5069	0.6212	0.4203	0.065*	
C7	−0.5839 (3)	0.4846 (3)	0.29260 (13)	0.0577 (5)	
H7	−0.7093	0.4768	0.2891	0.069*	
C8	−0.1882 (3)	0.7512 (3)	0.53408 (14)	0.0649 (6)	
H8	−0.3053	0.7513	0.5464	0.078*	
C9	0.0950 (2)	0.7909 (2)	0.54299 (11)	0.0460 (4)	
C10	0.2942 (2)	0.8537 (2)	0.58047 (11)	0.0448 (4)	
C11	0.4936 (2)	1.0475 (2)	0.71818 (12)	0.0464 (4)	
C12	0.6569 (2)	1.0709 (2)	0.68418 (13)	0.0538 (5)	
H12	0.6575	1.0310	0.6229	0.065*	
C13	0.8177 (3)	1.1539 (3)	0.74204 (14)	0.0599 (5)	
H13	0.9280	1.1682	0.7200	0.072*	
C14	0.8166 (3)	1.2157 (3)	0.83215 (14)	0.0629 (5)	
H14	0.9266	1.2713	0.8704	0.076*	
C15	0.6546 (3)	1.1965 (3)	0.86695 (13)	0.0572 (5)	
C16	0.4892 (2)	1.1100 (2)	0.80967 (12)	0.0506 (4)	
C17	0.6597 (3)	1.2699 (3)	0.96572 (15)	0.0823 (7)	
H17A	0.7840	1.3231	0.9941	0.124*	
H17B	0.6118	1.1762	0.9984	0.124*	
H17C	0.5864	1.3570	0.9675	0.124*	
C18	0.3094 (3)	1.0884 (3)	0.84471 (14)	0.0703 (6)	
H18A	0.2420	1.1706	0.8208	0.105*	
H18B	0.3330	1.1105	0.9108	0.105*	
H18C	0.2389	0.9703	0.8248	0.105*	

### Atomic displacement parameters (Å^2^)

**Table:**

	*U*^11^	*U*^22^	*U*^33^	*U*^12^	*U*^13^	*U*^23^
F1	0.0681 (9)	0.1287 (13)	0.1263 (13)	0.0247 (9)	−0.0216 (9)	−0.0270 (10)
F2	0.1052 (12)	0.1003 (11)	0.0926 (11)	−0.0021 (9)	−0.0311 (9)	−0.0318 (8)
O1	0.0631 (9)	0.0896 (11)	0.0523 (8)	0.0000 (8)	−0.0083 (7)	−0.0111 (7)
O2	0.0403 (7)	0.0849 (10)	0.0585 (8)	0.0131 (7)	0.0075 (6)	−0.0162 (7)
N1	0.0359 (8)	0.0537 (8)	0.0444 (8)	0.0091 (6)	0.0045 (6)	−0.0034 (6)
N2	0.0356 (8)	0.0569 (9)	0.0460 (8)	0.0084 (6)	0.0055 (6)	−0.0017 (7)
N3	0.0412 (9)	0.0964 (13)	0.0542 (10)	0.0167 (8)	0.0046 (7)	−0.0201 (9)
N4	0.0378 (8)	0.0640 (10)	0.0482 (9)	0.0110 (7)	0.0053 (6)	−0.0065 (7)
C1	0.0699 (15)	0.0672 (14)	0.0725 (15)	0.0012 (12)	−0.0212 (11)	−0.0079 (11)
C2	0.0521 (11)	0.0548 (11)	0.0466 (10)	0.0044 (8)	−0.0037 (8)	−0.0005 (8)
C3	0.0570 (12)	0.0698 (13)	0.0502 (11)	0.0090 (10)	0.0090 (9)	−0.0105 (9)
C4	0.0431 (10)	0.0625 (11)	0.0562 (11)	0.0076 (8)	0.0075 (8)	−0.0050 (9)
C5	0.0410 (9)	0.0456 (9)	0.0425 (9)	0.0072 (7)	0.0015 (7)	0.0009 (7)
C6	0.0436 (10)	0.0648 (12)	0.0495 (11)	0.0138 (9)	0.0027 (8)	−0.0050 (9)
C7	0.0410 (10)	0.0693 (12)	0.0582 (12)	0.0123 (9)	−0.0005 (8)	−0.0001 (9)
C8	0.0379 (10)	0.0943 (15)	0.0557 (11)	0.0151 (10)	0.0055 (8)	−0.0180 (10)
C9	0.0397 (9)	0.0541 (10)	0.0428 (9)	0.0117 (8)	0.0056 (7)	−0.0004 (8)
C10	0.0396 (9)	0.0511 (10)	0.0421 (9)	0.0100 (7)	0.0054 (7)	0.0012 (7)
C11	0.0413 (9)	0.0497 (10)	0.0451 (10)	0.0091 (7)	0.0030 (7)	0.0013 (8)
C12	0.0469 (10)	0.0614 (11)	0.0496 (10)	0.0084 (8)	0.0078 (8)	−0.0010 (8)
C13	0.0433 (10)	0.0661 (12)	0.0638 (12)	0.0047 (9)	0.0059 (9)	−0.0008 (10)
C14	0.0485 (11)	0.0651 (12)	0.0638 (13)	0.0056 (9)	−0.0084 (9)	−0.0033 (10)
C15	0.0601 (12)	0.0597 (11)	0.0472 (10)	0.0145 (9)	−0.0024 (9)	0.0008 (9)
C16	0.0514 (10)	0.0557 (11)	0.0436 (10)	0.0150 (8)	0.0037 (8)	0.0018 (8)
C17	0.0873 (17)	0.0976 (18)	0.0509 (12)	0.0169 (14)	−0.0047 (11)	−0.0101 (11)
C18	0.0616 (13)	0.0961 (16)	0.0510 (12)	0.0169 (11)	0.0128 (10)	−0.0033 (11)

### Geometric parameters (Å, °)

**Table:**

F1—C1	1.345 (3)	C6—C7	1.381 (2)
F2—C1	1.330 (3)	C6—H6	0.9300
O1—C1	1.346 (3)	C7—H7	0.9300
O1—C2	1.394 (2)	C8—H8	0.9300
O2—C10	1.215 (2)	C9—C10	1.487 (2)
N1—C8	1.341 (2)	C11—C12	1.387 (2)
N1—N2	1.3632 (19)	C11—C16	1.400 (2)
N1—C5	1.428 (2)	C12—C13	1.374 (3)
N2—C9	1.316 (2)	C12—H12	0.9300
N3—C8	1.314 (2)	C13—C14	1.374 (3)
N3—C9	1.355 (2)	C13—H13	0.9300
N4—C10	1.353 (2)	C14—C15	1.385 (3)
N4—C11	1.417 (2)	C14—H14	0.9300
N4—H4D	0.8600	C15—C16	1.402 (3)
C1—H1	0.9800	C15—C17	1.507 (3)
C2—C7	1.372 (3)	C16—C18	1.510 (3)
C2—C3	1.376 (3)	C17—H17A	0.9600
C3—C4	1.377 (3)	C17—H17B	0.9600
C3—H3	0.9300	C17—H17C	0.9600
C4—C5	1.380 (2)	C18—H18A	0.9600
C4—H4	0.9300	C18—H18B	0.9600
C5—C6	1.384 (2)	C18—H18C	0.9600
			
C1—O1—C2	118.10 (17)	N2—C9—N3	115.33 (15)
C8—N1—N2	109.18 (14)	N2—C9—C10	122.68 (15)
C8—N1—C5	129.19 (15)	N3—C9—C10	121.99 (15)
N2—N1—C5	121.60 (13)	O2—C10—N4	125.87 (16)
C9—N2—N1	102.16 (13)	O2—C10—C9	122.19 (15)
C8—N3—C9	102.47 (15)	N4—C10—C9	111.94 (14)
C10—N4—C11	129.00 (15)	C12—C11—C16	121.23 (16)
C10—N4—H4D	115.5	C12—C11—N4	120.80 (16)
C11—N4—H4D	115.5	C16—C11—N4	117.93 (15)
F2—C1—F1	105.50 (18)	C13—C12—C11	119.26 (17)
F2—C1—O1	106.7 (2)	C13—C12—H12	120.4
F1—C1—O1	110.9 (2)	C11—C12—H12	120.4
F2—C1—H1	111.2	C14—C13—C12	120.48 (18)
F1—C1—H1	111.2	C14—C13—H13	119.8
O1—C1—H1	111.2	C12—C13—H13	119.8
C7—C2—C3	120.57 (17)	C13—C14—C15	121.14 (18)
C7—C2—O1	123.26 (18)	C13—C14—H14	119.4
C3—C2—O1	116.09 (17)	C15—C14—H14	119.4
C2—C3—C4	120.04 (18)	C14—C15—C16	119.45 (18)
C2—C3—H3	120.0	C14—C15—C17	119.27 (19)
C4—C3—H3	120.0	C16—C15—C17	121.28 (19)
C3—C4—C5	119.59 (18)	C11—C16—C15	118.43 (17)
C3—C4—H4	120.2	C11—C16—C18	120.51 (16)
C5—C4—H4	120.2	C15—C16—C18	121.04 (17)
C4—C5—C6	120.32 (16)	C15—C17—H17A	109.5
C4—C5—N1	120.17 (15)	C15—C17—H17B	109.5
C6—C5—N1	119.50 (15)	H17A—C17—H17B	109.5
C7—C6—C5	119.65 (17)	C15—C17—H17C	109.5
C7—C6—H6	120.2	H17A—C17—H17C	109.5
C5—C6—H6	120.2	H17B—C17—H17C	109.5
C2—C7—C6	119.82 (18)	C16—C18—H18A	109.5
C2—C7—H7	120.1	C16—C18—H18B	109.5
C6—C7—H7	120.1	H18A—C18—H18B	109.5
N3—C8—N1	110.85 (16)	C16—C18—H18C	109.5
N3—C8—H8	124.6	H18A—C18—H18C	109.5
N1—C8—H8	124.6	H18B—C18—H18C	109.5
			
C8—N1—N2—C9	−0.5 (2)	C8—N3—C9—N2	−0.1 (2)
C5—N1—N2—C9	177.77 (15)	C8—N3—C9—C10	179.36 (18)
C2—O1—C1—F2	−162.99 (17)	C11—N4—C10—O2	−2.5 (3)
C2—O1—C1—F1	82.6 (2)	C11—N4—C10—C9	177.38 (16)
C1—O1—C2—C7	−37.5 (3)	N2—C9—C10—O2	−8.1 (3)
C1—O1—C2—C3	145.8 (2)	N3—C9—C10—O2	172.46 (18)
C7—C2—C3—C4	0.1 (3)	N2—C9—C10—N4	172.08 (16)
O1—C2—C3—C4	176.96 (18)	N3—C9—C10—N4	−7.4 (2)
C2—C3—C4—C5	−0.4 (3)	C10—N4—C11—C12	22.9 (3)
C3—C4—C5—C6	0.4 (3)	C10—N4—C11—C16	−159.57 (17)
C3—C4—C5—N1	−178.75 (17)	C16—C11—C12—C13	1.4 (3)
C8—N1—C5—C4	−178.5 (2)	N4—C11—C12—C13	178.84 (17)
N2—N1—C5—C4	3.6 (2)	C11—C12—C13—C14	−1.1 (3)
C8—N1—C5—C6	2.4 (3)	C12—C13—C14—C15	−0.1 (3)
N2—N1—C5—C6	−175.54 (16)	C13—C14—C15—C16	1.1 (3)
C4—C5—C6—C7	−0.2 (3)	C13—C14—C15—C17	−178.7 (2)
N1—C5—C6—C7	178.98 (16)	C12—C11—C16—C15	−0.4 (3)
C3—C2—C7—C6	0.1 (3)	N4—C11—C16—C15	−177.95 (16)
O1—C2—C7—C6	−176.50 (17)	C12—C11—C16—C18	178.06 (18)
C5—C6—C7—C2	−0.1 (3)	N4—C11—C16—C18	0.5 (3)
C9—N3—C8—N1	−0.2 (2)	C14—C15—C16—C11	−0.8 (3)
N2—N1—C8—N3	0.5 (2)	C17—C15—C16—C11	178.95 (19)
C5—N1—C8—N3	−177.64 (17)	C14—C15—C16—C18	−179.26 (19)
N1—N2—C9—N3	0.4 (2)	C17—C15—C16—C18	0.5 (3)
N1—N2—C9—C10	−179.09 (15)		

### Hydrogen-bond geometry (Å, °)

**Table:**

*D*—H···*A*	*D*—H	H···*A*	*D*···*A*	*D*—H···*A*
N4—H4D···N3	0.86	2.27	2.717 (2)	113
C6—H6···O2^i^	0.93	2.43	3.344 (2)	169
C8—H8···O2^i^	0.93	2.26	3.159 (2)	162

Symmetry codes: (i) *x*−1, *y*, *z*.
